# Supplementary material for: No evidence for causal effects of trust in science on intentions for health-related behavior
Source: Commun Psychol. 2025 Dec 11;4:7. doi: 10.1038/s44271-025-00375-7 (PMC12808179; doi:10.1038/s44271-025-00375-7)
Supplement: Supplementary file 2 — Supplementary Material [file 44271_2025_375_MOESM2_ESM.pdf]

Supplementary Materials

Supplementary Methods (Prestudy)

Testing the causal effects of trust in science on protective behavior during a pandemic requires an effective manipulation of trust in science in that context. In the prestudy, we thus developed such a manipulation and tested its effect on trust in general and on three dimensions of trust (expertise, integrity, and benevolence)[1].

Method

Participants and Design

The prestudy employed an experimental design. Participants were assigned either to the low trust or the high trust condition. The study was an online study distributed among students at a large German university in April 2021. Participants received course credits for participation. The final sample (after excluding 41 participants who failed a preregistered attention and conscientiousness check) consisted of 114 students (M_{age} = 24.0, SD_{age} = 6.5, 87 women and 27 men). Power analyses revealed that the sample size of 114 had a 75.26% power to detect medium effects (d = 0.5).

Procedure and Measures.

Trust in Science Manipulation.

Participants read ten real statements that actual German-speaking scientists gave during the COVID-19 pandemic. An explanation followed each statement in the style of a fact check that indicated whether the statement was correct or wrong. Notably, we manipulated the percentage of correct and wrong statements. Participants randomly assigned to the high trust condition were presented with mostly correct statements (i.e., 8 out of 10). In contrast, participants assigned to the low trust condition saw statements that were mostly wrong (8 out

of 10). The statements (see OSF materials for details) focused on expected epidemiological trends (e.g., waves, seasonality), typical symptoms of an infection, the dangerousness of COVID-19, and its transmissibility but were not related to preventive behaviors. Two example statements are presented in Supplemental Table 1.

Supplemental Table 1

Examples of high trust and low trust statements of German scientists.

Condition	Provided Statement	Provided Information About Correctness	Provided Source
High trust	*'It could be that the seasonality effect helps us in summer (...). We might get through the summer without too much trouble, but still have little population immunity and then run into a winter wave with an immunologically naive population. We need to prepare for that.'*	This statement turned out to be correct. The second corona wave with more than 20,000 new infections on many days in December 2020 was significantly higher in Germany than the first wave in April 2020	Virologist Christian Drosten, on 24.04.2020, in an interview with *ZIB2*
Low Trust	*'But we're not looking at the facts here either. Sars-CoV-2 is a droplet infection and not an airborne one.'*	This statement has turned out to be false. Droplets, but also aerosols (very fine airborne liquid particles and droplet nuclei), which can float in the air for a long time, play a role in the transmission of SARS-CoV-2.	Virologist Hendrik Streeck on 06.04.2020 in an interview with *Die Zeit*

Note. All statements were originally presented to participants in German and were translated by the authors using DeepL.

Measures.

Following Dohle and colleagues (2020)[2], who measured trust in science related to the pandemic, we measured trust in science using five items (e.g., "I trust German scientists to do what is right during the Corona crisis"; $\alpha = .90$; originally adapted from Nisbet and

colleagues[3]). In addition, we used the Muenster Epistemic Trustworthiness Inventory (METI) to measure different aspects of trust in science[1]. The METI consists of three subdimensions: expertise (measured with six items, α = .94), integrity (measured with four items, α = .86), and benevolence (measured with four items, α = .81). Answers were given on Likert scales ranging from 1 (e.g., honest) to 7 (e.g., dishonest) and we recorded the items so that higher values indicated higher levels of trust in science.

It should be noted that trust in science is operationalized here, as in previous research[2,4], as trust in scientists. Obviously, trust in science could also refer to other aspects, such as trust in scientific findings or the scientific method. However, we decided to focus on measures related to trust in scientists, which is most closely connected to our manipulation (scientists providing correct or incorrect statements).

Other measures. Participants completed a conscientiousness check, "In your honest opinion, should we use your data in our analyses in this study"[5] and a self-designed attention check. As an attention check, participants (re)read six statements from scientists and indicated for each statement whether they had seen it as part of the study materials, had not seen it, or did not know. Three statements were part of the low trust condition, and three were part of the high trust condition, which balanced the amount of known statements in both conditions. As preregistered, participants who answered four or more of these attention check questions incorrectly (including selecting "I don't know") were excluded from the analysis. Finally, we collected sociodemographic characteristics, including, among others, gender, age, and pandemic-related questions (e.g., vaccination status)

Supplementary Results (Prestudy)

Our results suggested that our manipulation was successful. Participants in the low trust condition indicated, on average, lower trust scores on the general trust scale (M = 4.8; SD = 1.2) compared to participants in the high trust condition (M = 5.6; SD = 1.0), t(112)

=3.90, $p < .001$, $d = 0.74$, 95%-CI[0.36, 1.12]. Likewise, participants in the high trust condition ascribed German scientists a higher expertise ($M = 5.7$; $SD = 1.1$), more integrity ($M = 5.5$; $SD = 1.0$), and higher benevolence ($M = 5.5$; $SD = 0.9$). Participants in the low trust condition, however, indicated lower trust, prescribing them lower expertise ($M = 5.0$; $SD = 1.2$)), $t(112) = 3.34$, $p = .001$, $d = 0.63$, 95%-CI[0.25, 1.01], less integrity ($M = 4.8$; $SD = 1.1$)), $t(112) = 3.67$, $p < .001$, $d = 0.69$, 95%-CI[0.31, 1.07], and less benevolence ($M = 5.0$; $SD = 1.0$), $t(112) = 2.63$, $p = .010$, $d = 0.50$, 95%-CI[0.12, 0.87]. Our manipulation thus had a substantial effect on all subscales of Henrik et al.'s (2015)[1] Trust Inventory, and not only on expertise, contrary to our prediction regarding the subscales.

Supplementary References

1. Hendriks, F., Kienhues, D. & Bromme, R. Measuring laypeople's trust in experts in a digital age: The Muenster Epistemic Trustworthiness Inventory (METI). *PloS One* **10**, e0139309 (2015).
2. Dohle, S., Wingen, T. & Schreiber, M. Acceptance and adoption of protective measures during the COVID-19 pandemic: The role of trust in politics and trust in science. *Soc. Psychol. Bull.* **15**, 1–23 (2020).
3. Nisbet, E. C., Cooper, K. E. & Garrett, R. K. The partisan brain: How dissonant science messages lead conservatives and liberals to (dis)trust science. *Ann. Am. Acad. Pol. Soc. Sci.* **658**, 36–66 (2015).
4. Wingen, T., Berkessel, J. B. & Englich, B. No replication, no trust? How low replicability influences trust in psychology. *Soc. Psychol. Personal. Sci.* **11**, (2020).
5. Meade, A. W. & Craig, S. B. Identifying careless responses in survey data. *Psychol. Methods* **17**, 437 (2012).
